# Cardiorespiratory Coordination after Training and Detraining. A Principal Component Analysis Approach

**DOI:** 10.3389/fphys.2016.00035

**Published:** 2016-02-12

**Authors:** Natàlia Balagué, Jacob González, Casimiro Javierre, Robert Hristovski, Daniel Aragonés, Juan Álamo, Oscar Niño, Josep L. Ventura

**Affiliations:** ^1^Institut Nacional d'Educació Fisica de Catalunya, Department of Health and Applied Sciences, University of BarcelonaBarcelona, Spain; ^2^Physiological Sciences II, L'Hospitalet de Llobregat, University of BarcelonaBarcelona, Spain; ^3^Faculty of Physical Education, Sport and Health, Ss. Cyril and Methodius University in SkopjeSkopje, Macedonia; ^4^Department of Education, Johannes Gutenberg-Universität MainzMainz, Germany

**Keywords:** cardiorespiratory exercise testing, complex adaptive systems, principal component analysis, coordinative variables, physiological variables, strength variables, training effects

## Abstract

Our purpose was to study the effects of different training modalities and detraining on cardiorespiratory coordination (CRC). Thirty-two young males were randomly assigned to four training groups: aerobic (AT), resistance (RT), aerobic plus resistance (AT + RT), and control (C). They were assessed before training, after training (6 weeks) and after detraining (3 weeks) by means of a graded maximal test. A principal component (PC) analysis of selected cardiovascular and cardiorespiratory variables was performed to evaluate CRC. The first PC (PC_1_) coefficient of congruence in the three conditions (before training, after training and after detraining) was compared between groups. Two PCs were identified in 81% of participants before the training period. After this period the number of PCs and the projection of the selected variables onto them changed only in the groups subject to a training programme. The PC_1_ coefficient of congruence was significantly lower in the training groups compared with the C group [*H*_(3, *N*=32)_ = 11.28; *p* = 0.01]. In conclusion, training produced changes in CRC, reflected by the change in the number of PCs and the congruence values of PC_1_. These changes may be more sensitive than the usually explored cardiorespiratory reserve, and they probably precede it.

## Introduction

Cardiorespiratory exercise testing is commonly used in clinical practice for both functional and diagnostic assessments of all types of populations. It measures a broad range of variables related to cardiorespiratory function with the goal of quantitatively linking metabolic, cardiovascular, and pulmonary responses to exercise (Balady et al., 2010). However, it provides little information about the coordinated activity of these subsystems. Although the study of cardiorespiratory coordination (CRC) is in its infancy and needs better assessment methods (Friedman et al., 2012), there is already some published evidence relating aging and disease with alterations in cardiorespiratory coupling (García et al., 2013; Iatsenko et al., 2013).

As a complex system the human organism acts as an indivisible and integrated whole that cannot be reduced to the quantitative analysis of its subsystem functions (West, 2006). Cardiovascular and cardiorespiratory subsystems are interdependent and interact in a dynamic and nonlinear way, that is, non-proportionally, and they therefore need to be approached through nonlinear models (Goushcha et al., 2014), the study of time series, and by using complex systems methodologies (Schulz et al., 2013) These methodologies, focusing on the coordinative aspects of human physiology, must be considered when studying the effects of training on cardiorespiratory function. Furthermore, as our organism tackles every new situation with an existing set of capabilities (Kelso, 1995) and continuously exchanges information with its environment, its behavior is unique and unexpected in the short term (weeks, months) (Hristovski et al., 2010), which is the usual duration of common training programmes.

In order to study the coordination between subsystems involving multiple variables, complex systems approaches propose the detection of coordination variables (the so-called order parameters or collective variables), because they capture the order or coordination of the whole system under study (Haken, 1983). Principal component analysis (PCA) is a common statistical technique that has been used to identify these coordination variables in a wide range of biological research fields including motor control (Newell and Vaillancourt, 2001), brain dynamics (Le Van Quyen, 2003), DNA replication (Elsawy et al., 2005), and protein folding (Maisuradze et al., 2009). PCA reduces the data dimension of coupled systems, extracting the smallest number of components that account for most of the variation in the original multivariate data and summarizing it with little loss of information. Although the PCA applied to kinematic variables has been successfully used to study the effects of motor learning processes (Newell and Vaillancourt, 2001), it has yet to be applied to study training effects on physiological variables. The PCA can be used to identify the degree to which time patterns of physiological responses co-vary, i.e., how much their increases and decreases are statistically synchronized. Two or more cardio-respiratory variables that co-vary in time may show different degree of co-variation: the more they co-vary the more variance they share and the more mutual information they create. This common variance and mutual information makes possible to represent the time patterns of single cardio-respiratory variables using fewer coordination variables (PCs). PCs are extracted in decreasing order of importance so that the first PC (PC_1_) accounts for as much of the variation as possible, with each successive component accounting for a little less. The number of PCs reflects the dimensionality of the system, such that a decrease in the number of PCs is indicative of greater coordination (fewer dimensions), and vice-versa. The number of PCs changes when the system suffers a qualitative or coordinative reconfiguration. Two aspects of the PCA can be usefully exploited. The first one may be called *pragmatic*, and consists of making the system under study easier to model by reducing the initial high dimension to significantly lower one (e.g., from six cardio-respiratory variables to one or two PCs). The second one is *explanatory* and consists of gaining insight in the degree of co-relatedness or its absence among the variables under study. Both aspects may present advantages over other methods for predicting the cardiovascular responses and outcomes.

Two main types of training programmes (aerobic, AT and resistance, RT) have been widely investigated due to their important and different physiological effects (Kenney et al., 2015). Their combination (AT + RT) has recently been recommended for health purposes in a wide variety of populations (Pollock et al., 2000).

Given that cardiorespiratory function cannot be tested solely through quantitative measures, and since there are no studies evaluating the effects of training programmes and detraining on CRC, we aim to investigate the qualitative changes in CRC in healthy young men before and after a period of 6 weeks of different training modalities (AT, RT, and AT + RT), and then again 3 weeks after detraining. We specifically hypothesize (1) that training will produce a reduction in the number of PCs, in other words, that it will improve CRC, and (2) that the qualitative information obtained through our approach will complement the quantitative data that are usually obtained.

## Materials and methods

### Participants

Thirty-two healthy, physically active males (age 21.2 ± 2.4 years, height 177.1 ± 6.6 cm, mean body mass 71.0 ± 5.1 kg and mean body mass index 22.6 ± 1.7 kg· m^−2^), all physical education students with no sport specialization but who engaged in a wide range of aerobic activities at least three times a week, volunteered to participate in the study. After the baseline tests they were randomly assigned to four groups for the 6 weeks of training: aerobic (AT), resistance (RT), aerobic + resistance (AT + RT), and control (C), without changes in physical activity.

### Procedure

Participants completed a standard medical questionnaire in order to confirm their healthy status and level of physical activity. They also signed an informed consent form. All experimental procedures were approved by the Bio-ethics commission of Barcelona University. After the baseline cardiorespiratory testing and maximal strength and power tests (see below), they followed their assigned specific training programme three times a week:

1. AT group (*n* = 8): participants pedaled 60min at 60% of their individual maximum workload (60% W_max_). This workload was increased by 5% weekly unless the participant was unable to maintain the pace throughout the session. Heart rate was monitored during all the sessions.
2. RT group (*n* = 8): participants performed a 30-min strength circuit twice (Pollock et al., 2000). Starting weights were 40% of 1RM for the upper body [i.e., squat, chest press, shoulder press, triceps extension, biceps curl, and pull-down (upper back)] and 60% for the lower body [quadriceps extension, leg press, leg curls (hamstrings), and calf raise]. Participants were allowed a maximum of 12 repetitions, which included a slow controlled movement (2 s up and 4 s down). The resting period between exercises was 2 min. Workloads were adjusted weekly, with resistance being increased as needed (typically 5–10%) if the participant was able to lift the weight comfortably (i.e., more than 12 repetitions).
3. AT + RT group (*n* = 8): participants pedaled at 60% W_max_ for 30 min and performed the strength circuit (same as the R group) once.
4. C group (*n* = 8): control participants continued with their usual activities, without any special training.

### Cardiorespiratory testing

The incremental cycling test (Excalibur, Lode, Groningen, Netherlands) started at 0 W and the workload was increased by 20 W/min until participants could not maintain the prescribed cycling frequency of 70 rpm for more than 5 consecutive seconds. During the test, participants breathed through a valve (Hans Rudolph, 2700, Kansas City, MO, USA) and respiratory gas exchange was determined using an automated open-circuit system (Metasys, Brainware, La Valette, France). Oxygen and CO_2_ content and air flow rate were recorded breath by breath. Before each trial, the system was calibrated with a mixture of O_2_ and CO_2_ of known composition (O_2_ 15%, CO_2_ 5%, N_2_ balanced) (Carburos Metálicos, Barcelona, Spain), as well as with ambient air.

Haemodynamic information was obtained from participants using non-invasive finger cuff technology (Nexfin, BMEYE Amsterdam, Netherlands). The Nexfin device provides continuous blood pressure (BP) monitoring from the resulting pulse pressure waveform, and calculates both systolic and diastolic blood pressure (SBP and DBP). Participants were connected by wrapping an inflatable cuff around the middle phalanx of the finger. The finger artery pulsing is “fixed” to a constant volume by application of an equivalent change in pressure against the blood pressure, resulting in a waveform of the pressure (clamp volume method). The measurement was performed in the non-dominant arm; relaxed, supported by a measurement cable attached by a rubber band. We enabled continuous monitoring finger photoplethysmography, which is useful for assessing acute BP changes (Eckert and Hornskotte, 2002). Electrocardiogram (ECG) was continuously monitored (DMS Systems, DMS-BTT wireless Bluetooth ECG transmitter, and receiver, software DMS Version 4.0, Beijing, China).

All tests were performed in a well-ventilated lab; the room temperature was 23°C and the relative humidity 48%, with variations of no more than 1°C in temperature and 10% in relative humidity. The tests were carried out at least 3 h after a light meal, and participants were instructed not to perform any vigorous physical activity for 72 h before testing. Participants repeated the tests after6 weeks of training and again after 3 weeks of detraining.

### Maximal strength and power testing

Maximal strength and maximal power of upper and lower limbs, respectively, were measured (Musclelab Power System, Porsgruun, Norway) in each participant. Estimated 1 RM-chest press and 1RM-squat based on submaximal loads was calculated. In the chest press exercise the load started with 25 kg and continued with 35, 45, 55, 65 kg, etc. In the squat exercise, participants started with 45 kg and continued with 65, 85, 105 kg, etc. until they were unable to perform one repetition. Based on these results the maximal 1RM was registered and the force/velocity relationship graph was plotted to determine the maximal power.

All exercise tests were carried out at least 3 h after a light meal and participants were instructed not to perform any vigorous physical activity for 72 h before testing. Participants repeated these tests after 6 weeks of training and again after 3 weeks of detraining.

### Data analysis

The following maximal values of performance and cardiorespiratory variables were registered during the tests: ventilatory threshold in % of VO_2max_ (by means of the O_2_ and CO_2_ ventilatory equivalents method, Reinhard et al., 1979), maximal cycling workload (Wmax), maximal oxygen uptake relative to body weight (VO_2_/kg.min max), maximal expiratory ventilation per minute (VE_max_), maximal heart rate (HR_max_), maximal 1RM-squat, and maximal 1RM-chest. The group means in the different conditions were compared using the non-parametric Friedman ANOVA.

**Figure 1:**
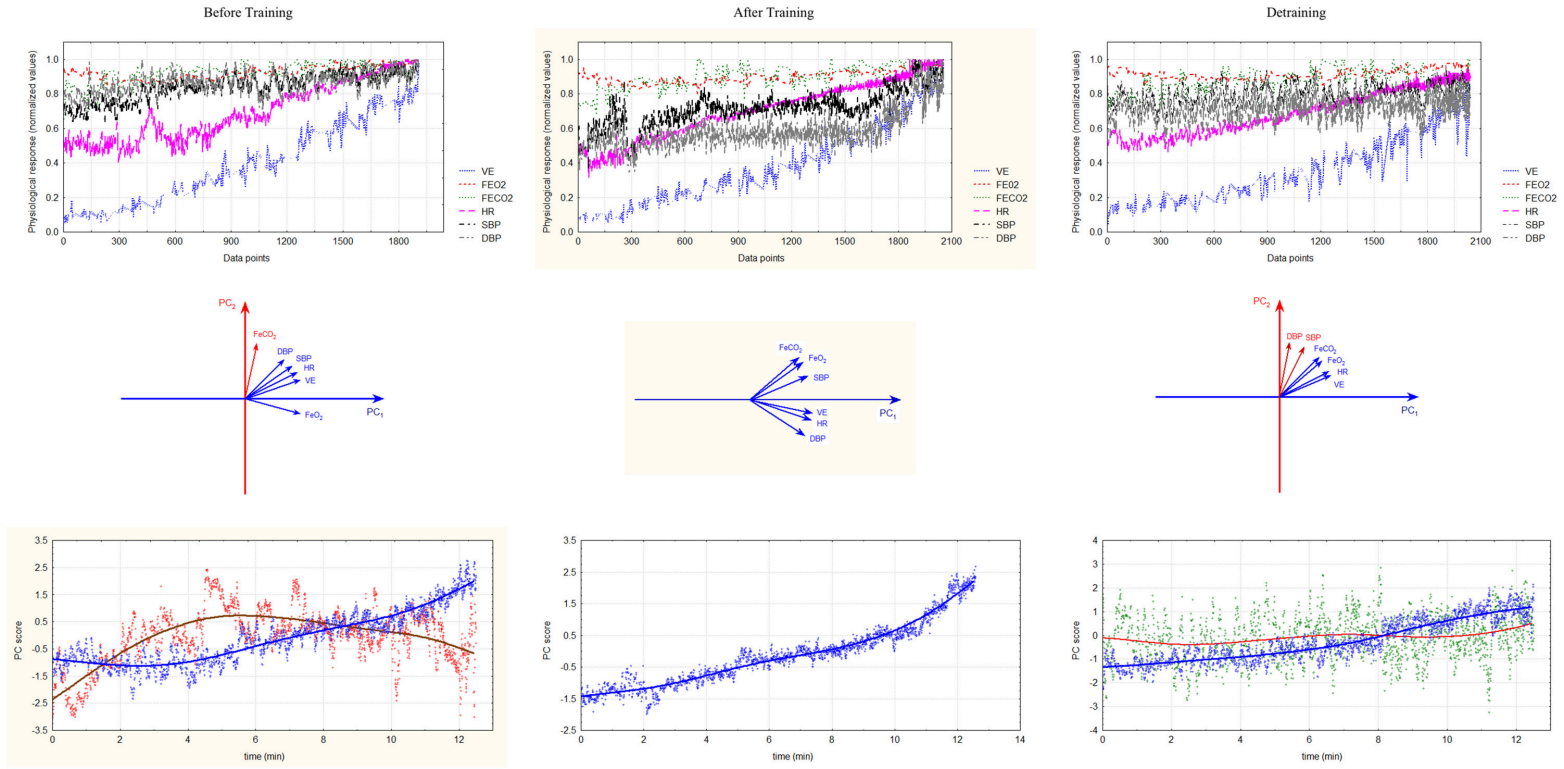
**Typical example of the reduction of cardio-respiratory variables to time series of cardio-respiratory coordination variables or PCs (from up to down) and their changes with training and detraining (from left to right)**. Upper graphs: Original time series of the six measured cardio-respiratory variables in the three conditions (before and after training and detraining). Middle graphs: Positions of the six original cardio-respiratory vectors within the coordinate system of PCs. The colors of unit vectors correspond to PCs they form. Lowest graphs: Time series of PC scores (standardized *z*-values in the space spanned by PCs). Note that the six time series are collapsed to only one or two time series as a consequence of the PC dimension reduction. The blue and the red line show the average trend of both processes as calculated by weighted least squares method. The number of PCs decreases after training and increases after detraining.

In order to study the CRC in each participant a PCA was performed on the time series of the following selected cardiorespiratory variables: expired fraction of O_2_ (FeO_2_), expired fraction of CO_2_ (FeCO_2_), ventilation (VE), systolic blood pressure (SBP), diastolic blood pressure (DBP), and heart rate (HR). Other commonly registered variables in cardiorespiratory testing such as respiratory equivalents, respiratory exchange ratio, oxygen pulse, oxygen consumption, etc. were excluded from the analysis due to their known deterministic mathematical relation with the aforementioned variables. Figure 1 shows a typical example of how the PCA reduces the dimensionality of the six measured cardio-respiratory variables into one or two coordinative variables (PCs). The upper graphs display the time series of the six selected variables, the middle graphs show the degree of unit vector co-linearity of the standardized variables on PC_1_ and PC_2_ and the lower graphs the time series of the PCs. As it can be seen there is a reduction in dimensionality from six cardio-respiratory variables (upper graphs) to one or two PCs (lowest graphs). In the middle graphs, unit vectors of the six variables mainly project to, that is, align with, a certain PC. The blue and red lines show the average trends of the two processes (weighted using the least squares method). Correlations r of unit vectors with PCs are given as *r* = cos α; where α is the angle between the unit vectors and the PCs.

The number of generalizable PCs was determined by the Kaiser-Gutmann criterion, which treats as significant the PCs with eigenvalues λ ≥ 1.00 (Jolliffe, 2002). The optimal parsimony solution of the extracted PCs was obtained by the Varimax orthogonal rotation criterion (Meglen, 1991). In order to compare the structure of the extracted PCs before and after training and after the detraining period we used Tucker's congruence coefficient, which is Salton's cosine similarity measure applied to PCs (Lorenzo-Seva and Ten Berge, 2006).

Since the first PC always contains the largest proportion of the data variance we estimated the congruence coefficients only between the first PCs within each group for the three different conditions (pre, post training, and post detraining condition). The median of the PC_1_ coefficient of congruence was obtained in each group and condition (before training and after both training and detraining). The null hypothesis of a constant PC congruence median across the control group and the training groups was tested by means of a non-parametric Kruskal-Wallis ANOVA. Mann-Whitney U matched pairs test analysis was also performed to assess statistically significant differences between each pair of different conditions. Effect sizes (Cohen's *d*) were computed to demonstrate the magnitude of standardized differences in medians where effects reached the *p* < 0.05 level.

## Results

Table 1 shows the mean and SD of the ventilatory threshold, maximal workload, cardiorespiratory and strength variables registered in the three situations (before training and after both training and detraining) in all groups, not detecting the ANOVA significant differences. Only the AT + RT group showed significant changes in W_max_ after the training period (*p* = 0.02). Cohen's *d*-values were 0.7 between the conditions “before training” and “after training” and between “after training” and “after detraining” in this group.

**Table 1:** **Means (standard deviations) of ventilatory threshold, maximal workload, and maximal cardiorespiratory and strength variables**.

**Group**	**Condition**	**VEmax (l·min^−1^)**	**VO_2_max (ml·Kg·min^−1^)**	**VTh (%VO_2_max)**	**HRmax (b·min^−1^)**	**W max (W)**	**Chest press max (kg)**	**Squat max (kg)**
AT	1	125.5	51.3	40.5	185.8	301.9	62.8	167.8
		(28)	(9.7)	(2.8)	(10.7)	(17.4)	(6.3)	(29.2)
	2	149.8	60.1	47.4	184.2	328.7	68.5	166.4
		(34.8)	(9.7)	(3.2)	(16.9)	(13.3)	(4.7)	(25.4)
	3	137.1	57.5	44.2	182.6	312.8	71.4	165.7
		(25.1)	(9.1)	(4.3)	(15.6)	(16.5)	(4.7)	(30.3)
RT	1	119	47.3	36.8	183.9	270.2	63.3	147.5
		(22.9)	(6.4)	(1.8)	(12.5)	(23.4)	(5.7)	(16.6)
	2	124.8	49.6	41.4	177.9	273	72.5	165.6
		(22.5)	(7.7)	(2.9)	(14.3)	(23.5)	(5.3)	(16.5)
	3	117.9	50.3	36.6	177.0	267.8	72.5	151.2
		(23.8)	(5.2)	(1.5)	(12.9)	(19.6)	(9.2)	(24.8)
AT + RT	1	117.8	45.1	40.3	175.9	273.3	71.6	156.1
		(15.7)	(5.3)	(4.3)	(10)	(12.7)	(19.5)	(24.7)
	2	123.1	49.6	39.3	171.9	302.6	76.6	158.3
		(19.6)	(3.8)	(2.4)	(9.8)	(7.5)	(16.3)	(24.8)
	3	125.9	51.2	38.9	173.5	290.	81.6	158.8
		(19.8)	(8.1)	3	(12.1)	(8.9)	(15.8)	(24)
C	1	125.7	51.2	37.8	184.6	280.8	67.2	167.2
		(24.8)	(7.8)	(2.8)	(9.3)	(28.8)	(10.3)	(27.2)
	2	120.2	50.5	44.3	175.3	278.2	73.3	160
		(24.8)	(7.4)	(3.5)	(7.7)	(26.7)	(10.3)	(30.3)
	3	123.6	49.4	38.5	177.1	277.4	76.1	148.7
		(25.3)	(6.9)	(2.6)	(10.2)	(32.5)	(10.5)	(34.6)

*VEmax, maximal expired minute volume; VO_2_max., maximal oxygen uptake relative to body weight; VTh, ventilatory threshold; HRmax., maximal heart rate; Wmax., maximal cycling workload; AT, aerobic training; RT, resistance training; AT + RT, mixed training; C, control; 1, before training; 2, after training; 3, after detraining*.

A typical result of the effect of the three conditions (before training, after training, and detraining) on the CRC is shown on Figure 1. In the left column (before training) we see that five variables (VE, HR, FeO_2_, SBP, and DBP) show a larger degree of co-linearity with PC_1_ and that only FeCO_2_ is dominantly aligned with PC_2_. Thus, PC_1_ represents the CRC and PC_2_ the idiosyncratic behavior of FeCO_2_. After training (middle column) the variance of the six cardio-respiratory variables is captured by a sole PC_1_. This is because the unit vectors of all variables, including FeCO_2_, are more closely aligned with PC_1_. Note that it is the shift of the FeCO_2_ toward the PC_1_ cluster of variables that enables the formation of this sole PC_1_. After detraining (right column) the cardio-respiratory variables again form two PCs, pointing to a possible decrease in the coordinative tendencies. This time SBP and DBP are projected in PC_2_.

**Figure 2:**
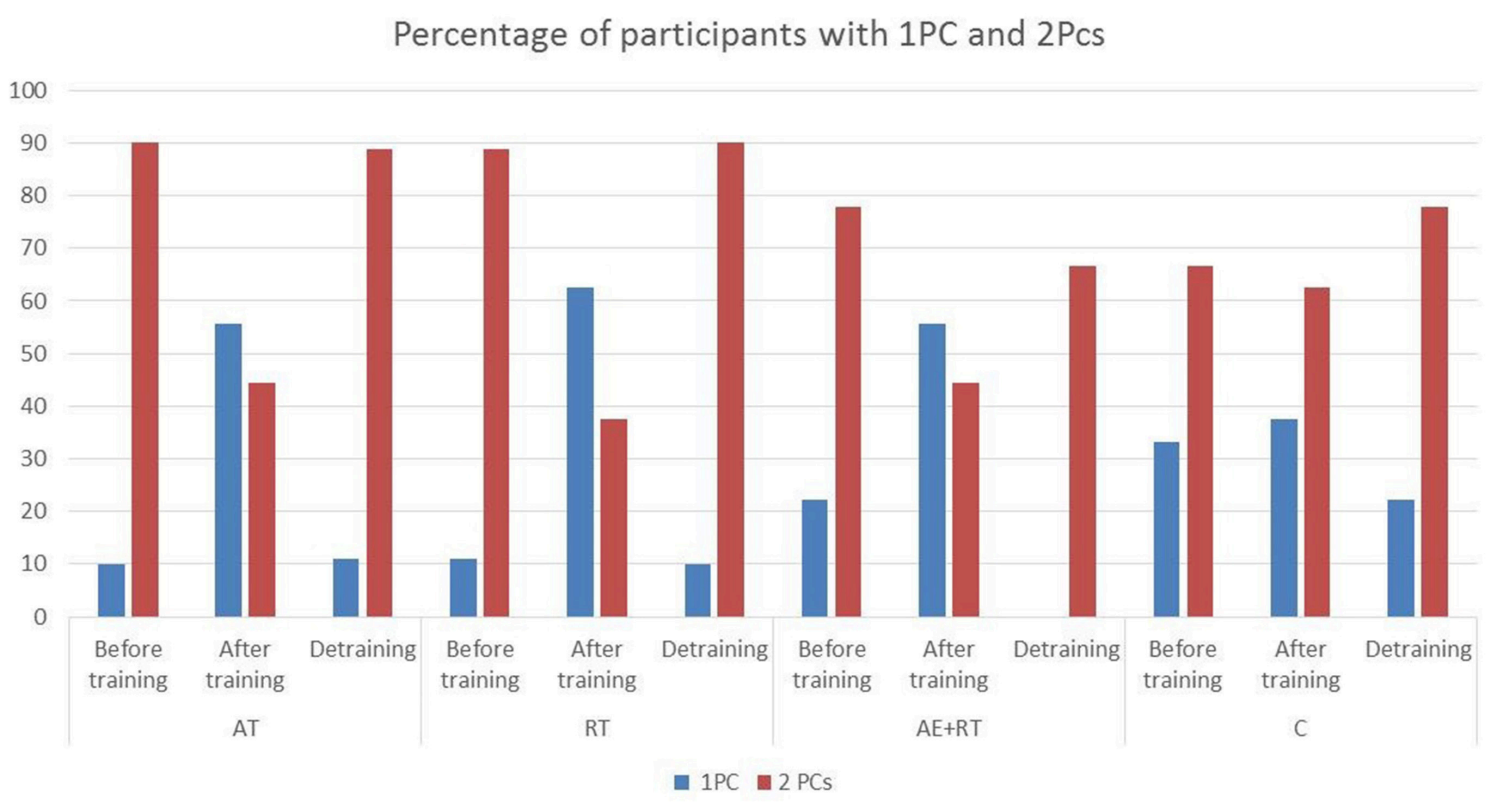
**Percentage of participants with one PC (PC_1_) and two PCs (PC_1_ and PC_2_) in the three conditions in all groups**. AT, aerobic training; RT, resistance training; AT + RT, mixed training; C, control; 1, before training; 2, after training; 3, after detraining. In RT group one participant had three PCs before training and after detraining and in AT + RT group one participant had three PCs after detraining.

**Figure 3:**
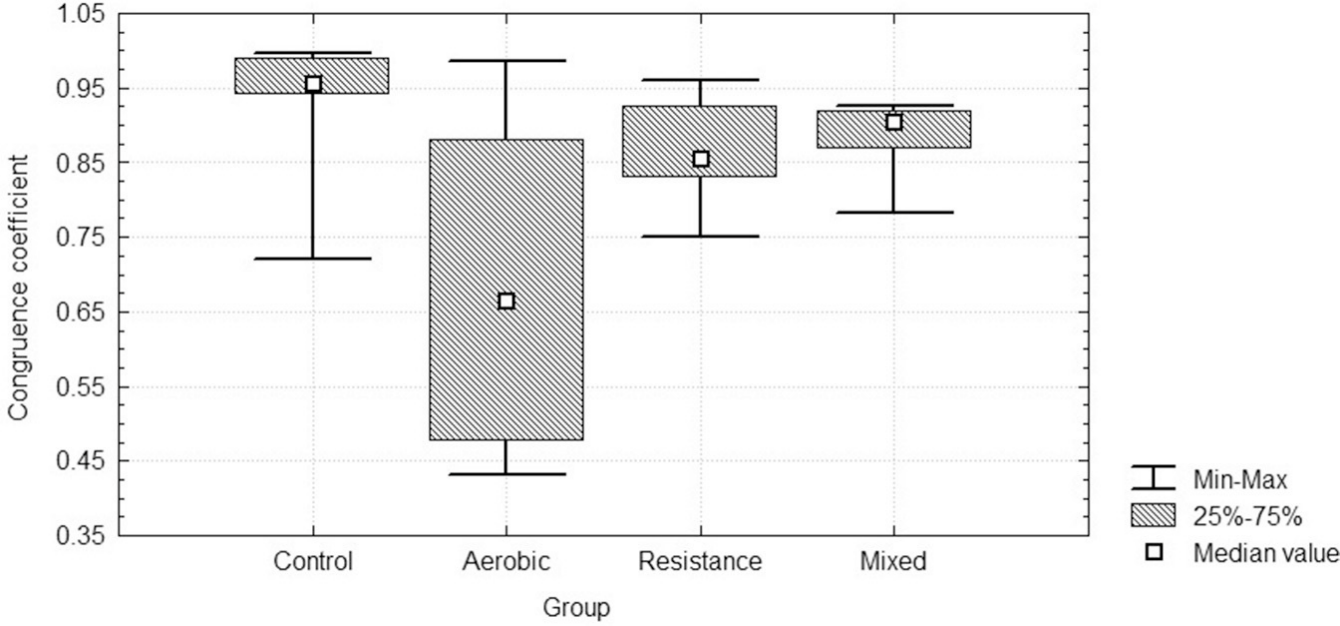
**Medians of the PC_1_coefficient of congruence between the three conditions (before training and after both training and detraining) in all groups**.

Figure 2 shows the percentage of participants having one PC (PC_1_) or two PCs (PC_1_ and PC_2_) in the three conditions in each experimental group. Before training two PCs where found in 81.3% of participants and a single PC in 16.6 % of participants. Four variables (VE, HR, SBP, DBP) plus FeCO_2_ or, alternatively, FeO_2_ were always involved in forming PC_1_, whereas PC_2_ was formed by a single variable: either FeCO_2_ or FeO_2_. The number of PCs was reduced after training in the AT, RT, and AT + RT groups (54% of participants had two PCs and 45.8 had one PC), returning to initial values after detraining, whereas no changes were observed in the C group. Table 2 shows the mean and SD of the loading of the selected cardiorespiratory variables onto PC_1_ in the three conditions inall groups. PC_1_ was saturated by the loading of FeO_2_, VE, SBP, DBP, and HR, while PC_2_ was saturated bythe projection of FeCO_2_ or, alternatively, by FeO_2._ The eigenvalues of PC_1_ increased significantly in RT [χ${}_{\left(8,2\right)}^{2}$ = 10.75, *p* = 0.005] and AT + RT [χ${}_{\left(8,2\right)}^{2}$ = 1.56, *p* = 0.003] but not in the AT (with large SD) and C groups (see Table 3). The medians of the PC_1_ coefficient of congruence between the three conditions (before training and after both training and detraining) in the different groups are compared in Figure 3. The size of the PC_1_coefficient of congruence was lower in the groups subject to training, as compared with the C group [*H*_(3, *N*=32)_ = 11.28; *p* = 0.01], with large effect sizes between C and AT (*d* = 1.5), C and RT (*d* = 0.8), and C and AT + RT (*d* = 0.7).

**Table 2:** **Projection of the selected cardiovascular and cardiorespiratory variables onto PC_1_ in all participants**.

**Variables**	**Before training**		**After training**		**After detraining**	
	***M***	***SD***	***M***	***SD***	***M***	***SD***
VE	0.79	0.08	0.93	0.02	0.66	0.28
FeO_2_	0.52	0.10	0.63	0.14	0.47	0.25
FeCO_2_	0.64	0.15	0.77	0.04	0.57	0.29
HR	0.91	0.04	0.93	0.03	0.79	0.21
SBP	0.82	0.03	0.87	0.03	0.78	0.19
DBP	0.75	0.09	0.80	0.04	0.76	0.19

*VE, expired minute volume; FeO_2_, expired fraction of O_2_; FeCO_2_, expired fraction of CO_2_; HR, heart rate; SBP, systolic blood pressure; DBP, diastolic blood pressure*.

**Table 3:** **Eigenvalues of PC_1_ in the three conditions in all groups**.

**Group**	**Before training**		**After training**		**After detraining**	
	***M***	***SD***	***M***	***SD***	***M***	***SD***
AT	3.62	0.61	4.48	0.76	3.24	0.45
RT	3.51	0.46	4.12	0.53	3.13	0.13
AT + RT^*^	3.78	0.73	4.26	0.27	3.05	0.03
C	3.95	0.68	4.23	0.47	4.07	0.36

*AT, aerobic training; RT, resistance training; AT + RT, mixed training; C, control*.

* *p < 0.05*.

## Discussion

The present study was conceived to examine coordinative dimensional changes in cardiorespiratory testing results after a period of training and 3 weeks after detraining. Our hypothesis was based on two assumptions: (a) complex adaptive systems behave as a whole and thus they cannot be evaluated solely through quantitative measurements of isolated subsystem variables, and (b) PCA, providing information about the dimensionality of CRC, is able to capture the qualitative changes produced by training and detraining. Accordingly, our method of assessing CRC was able to provide qualitative information that complements the usually tested quantitative cardiorespiratory reserve and maximal performance values. Our findings indicated that: (a) the selected cardiorespiratory variables (FeO_2_, FeCO_2_, VE, SBP, DBP, and HR) obtained through an incremental cardiorespiratory test were reduced in 81.2% of participants to two dimensions (PC_1_ and PC_2_), summarizing the integrated CRC in young trained males before training; (b) only in the training groups was the number of PCs reduced after training, with the initial dimensionality being recovered after detraining; (c) the variables loading onto PC_1_ remained constant in the C group but changed in different ways after training and detraining under the different training modalities; (d) the PC_1_ coefficient of congruence after both training and detraining was significantly lower in the training groups compared with the C group; and (e) the dimensionality reduction in the CRC seemed to precede the quantitative physiological and performance changes that usually follow training and detraining.

Although most of the variables projected onto PC_1_ were highly correlated (see Table 2) they did not show perfect coupling with the dimension, in other words, none of them totally explained PC_1_. Only in the training groups did the projected variables and their correlation values change after the training intervention, indicating that a coordinative, nonlinear reconfiguration occurred after training. It is worth noting that the term “nonlinear” used in this paper does not refer to a non-linear fitting (e.g., exponential or logarithmic) of a theoretical curve to the data, but rather to a qualitative change in the data series. Thus, CRC is not rigid, but rather flexible and adaptive to changing requirements (Kelso, 1995).

The concept of CRC used here should be distinguished from the concept of “cardiorespiratory coupling,” which refers to the adjustment of heart beats at phases of the respiratory cycle (respiratory sinus arrhythmia). CRC assumes a mutual influence of cardiovascular and respiratory oscillations leading to spontaneous coordination.

The current findings reveal that the main coordinative variable (PC_1_) was loaded by all selected variables except FeO_2_ or, alternatively, FeCO_2_, which were included in PC_2_. This means that FeCO_2_ and FeO_2_ were less correlated with the remaining variables. In fact, while VE, HR, SBP, and DBP values increased with workload during the incremental test, FeCO_2_ and FeO_2_ followed a differentiated pattern of behavior. As has been previously described, FeCO_2_ increases at exercise onset and decreases approaching exhaustion, while FeO_2_ follows the contrary pattern (Skinner and McLellan, 1980). The duration of the decreasing FeCO_2_ and FeO_2_ phases probably determines whether a new PC (PC_2_) is formed or not. As is known, the decrease in FeCO2 at the end of an increasing exercise test is a consequence of the hyperventilation produced by the decrease in blood pH. Thus, if such decrease is manifested less in the data series (i.e., it affects fewer data points) for the same or higher workload this means that the ventilation and/or the buffering are more efficient. After 6 weeks of training, most participants (especially those in the AT group) reduced their initial number of PCs (from 2 PCs to 1 PC). This means that the set of studied variables improved their degree of co-variation and all aligned with PC_1_. A dimension reduction is a hallmark of formation of coordinative structures (Kelso, 1995), and so, the decrease in the number of PCs can be interpreted as an improvement in the efficiency of the CRC. This was probably due to a greater efficiency of gas exchange, as has been shown when studying the synchronization between heartbeat and respiration (Ben-Tal et al., 2012). The possible improved efficiency of gas exchange was not reflected in quantitative changes of the ventilatory threshold obtained through the ventilatory equivalents method. However, the relationship between PCA changes and the so-called ventilatory threshold (Reinhard et al., 1979) should be specifically investigated. In fact, the PCA, applied for the first time to the results obtained in cardiorespiratory fitness tests, could help to clarify the controversies around the detection of this threshold.

Although CRC improved after training no significant quantitative increases in cardiorespiratory reserve or performance (ventilatory threshold, VO_2max_, W_max_ and maximal strength and power tests) were observed in the groups under study (see Table 1). Some authors have likewise reported mild or no physiological and performance changes after 6 weeks of aerobic and resistance training in moderately trained participants (Niño et al., 2014) The previous fitness status, combined with the short training period, may explain the negative results obtained here. This means that the CRC assessment may be more sensitive to training and precede the commonly registered quantitative changes. It may also help to discern if, in some cases, the quantitative changes are attributable to training or to other effects (Katch et al., 1982).

The reversibility of the CRC changes with detraining in the AT, RT, and AT + RT groups increased the consistency of the findings. However, these preliminary results need to be confirmed with further research. From a methodological point of view the PCA is a linear dimension reduction technique and consequently it is sensitive only to linear correlational structure within the data. In future research it might be interesting, and even desirable, to apply other statistical techniques for dimension reduction that are more general with this respect, especially its nonlinear generalization such as nonlinear PCA methods (Tenenbaum et al., 2000) or network component analysis (NCA) (Liao et al., 2003). The use of these methods may capture more accurately the curvilinear covariations of physiological signals and hence account for more subtle coordinative tendencies. For example, although the hierarchical PCA is similar to NCA in avoiding orthogonality assumptions about the eigenvectors (PCs), it offers an additional advantage of faithfully reconstructing the network topology when only a partial knowledge is available. The use of these methods in exercise physiology may prove to be fruitful for understanding the intricacies of organic interactions at macroscopic level. From a clinical point of view further research is warranted to clarify (1) if the number of PCs is related to health or training status, (2) if their reduction is linked to better physiological adaptation and performance or health prognosis, (3) the nature of the relationship between CRC and the ventilatory threshold, and (4) the effects of other training programmes such as high-intensity or intervallic training on CRC. As cardiorespiratory exercise testing is applied to a wide range of populations of all ages and training status, future studies should test CRC in athletes of different levels, and also in clinical patients, especially those with cardiorespiratory diseases. Improving the interpretation of cardiorespiratory exercise testing is crucial for healthcare and for wellbeing in general.

The current results show the sensitivity of CRC to training and detraining and highlight the value of incorporating complex systems approaches into the current strategic research framework for sport and exercise medicine (Holtzhausen et al., 2014). However, a longer term study would be required to test the sensitivity of CRC assessments.

## Author contributions

NB, CJ, RH, and JV conceived the paper and jointly drafted and reviewed the content; RH conceived the approach to data analysis; JG, DA, JA, and ON worked on acquisition and analysis of the data. The authors approved the final version and agree to be accountable for all aspect of the work. Conception and design of the work.

## Funding

This study has been supported by the Institut Nacional d'Educació Física de Catalunya (INEFC), Generalitat de Catalunya.

### Conflict of interest statement

The authors declare that the research was conducted in the absence of any commercial or financial relationships that could be construed as a potential conflict of interest.
